# Influence of the Culinary Treatment on the Quality of *Lactarius deliciosus*

**DOI:** 10.3390/foods2020238

**Published:** 2013-06-17

**Authors:** Krystyna Pogoń, Grażyna Jaworska, Aleksandra Duda-Chodak, Ireneusz Maciejaszek

**Affiliations:** 1 Department of Raw Material and Processing of Fruit and Vegetables, University of Agriculture in Krakow, Balicka 122, 30-149 Krakow, Poland; E-Mail: rrgjawor@cyf-kr.edu.pl; 2 Department of Fermentation Technology and Technical Microbiology, University of Agriculture in Krakow, Balicka 122, 30-149 Krakow, Poland; E-Mail: aduda-chodak@ar.krakow.pl; 3 Department of Refrigeration and Food Concentrates, University of Agriculture in Krakow, Balicka 122, 30-149 Krakow, Poland; E-Mail: rrmaciej@cyf-kr.edu.pl

**Keywords:** mushrooms, *Lactarius deliciosus*, frying, antioxidant activity, proximate composition, sensory propertie

## Abstract

The influence of culinary treatment on the nutritional value and quality of *Lactarius deliciosus* was established. Mushrooms: unblanched (I), blanched (II), and unblanched with onion and spices (III), were fried in oil for 10 min. Fried mushrooms were assessed before storage as well as after 48 h in storage at 20 °C, and after 48 and 96 h in storage at 4 °C. Frying increased the dry weight, protein, fat, ash, total carbohydrate, total polyphenol, and total flavonoid content, as well as the caloric value of the mushrooms. In addition, frying decreased the antioxidant activity, color parameters (a*, h*), and texture. The most significant changes due to culinary treatment and storage were observed in type II product. Microbiological analysis of the samples after a 48 h storage period at 20 °C revealed the total viable count over 10^6^ and contamination with lactic acid bacteria. Fried mushrooms stored at 4 °C for 96 h were free from microorganisms.

## 1. Introductions

Among 2000 species of mushrooms only over a dozen are consumed by people and consumers consider wild mushrooms to be more attractive than cultivated ones [1,2]. *Lactarius deliciosus* are one of the most popular mushrooms because of their unique sensory characteristics. Not only are they a source of basic nutrients [1], but also a source of antioxidant compounds like polyphenols [3,4], and particular polyol compounds and phenylpropanoid glycosides like lactaroviolin which are found only in this particular species of edible mushroom [5]. Entering *Lactarius deliciosus* in the diet may have a positive influence on health as a result of anti-inflammatory activity and hypocholersterolemic activity attributed to the mushrooms [6].

Frying as a method of culinary treatment influences the characteristics of food products, particularly their nutritional value and their sensory characteristics. A decrease is observed in the level of nutrients such as unsaturated fatty acids, minerals, and vitamins. At the same time, thermal treatment increases the digestibility of proteins, causes starch gelatinization, and produces specific sensory characteristics as a result of the Maillard reaction, as well as texture characteristics specific to the products [7,8,9]. According to Bognar [7], among other methods of culinary treatment, frying causes the smallest decrease in valuable nutrients. In Poland and in Europe, the traditional way of preparing *Lactarius deliciosus* mushrooms for consumption is to fry the fruiting bodies in oil or butter, and onion and other spices are also added. The influence of the process on nutritional value and quality characteristics of the mushrooms has not yet been researched, however information on the composition of fresh mushroom is available [2]. 

The objective of this paper was to determine the influence of culinary treatment and of storage conditions on the quality of the *Lactarius deliciosus* mushrooms. Quality assessment included the proximate composition, antioxidant properties, color, texture, as well as sensory and microbiological analysis.

## 2. Experimental Section

### 2.1. Material

The materials were the caps of *Lactarius deliciosus* (L.) Pers. mushrooms and the culinary products prepared with them.

The mushrooms were purchased from a wild mushrooms salesman, certified as an expert. The fruiting bodies were fresh, healthy, and of similar size with their cap diameter between 3 and 6 cm. Culinary treatment was conducted approximately 10 h after the mushrooms had been picked. 

Damaged and infested fruiting bodies were disposed off and the remaining bodies were rinsed in cold water and cut into cubes of a 1 cm width. One third of the mushroom caps were also blanched in a 0.5% solution of citric acid (98 °C/90 s) in ratio 1000 g of mushroom caps per 5 L of blanching solution. After the initial preparation, the mushroom caps were subdued to culinary treatment, consisting of frying in a small amount of oil. Frying was conducted in a Teflon pan and under a cover at 100 °C.

Three types of culinary products were prepared:

- Product prepared with unblanched mushroom caps,
- Product prepared with blanched mushroom caps,
- Product prepared with unblanched mushroom caps with the following ingredients (quantity per 1 kg of mushrooms): 100 g of onion, 10 g of garlic, 20 g of ground black pepper, 1 g of allspice grains, and 1 g of bay leaf.

After conducting frying, per 1000 g of material (mushroom caps and additions), the following quantities of fried mushrooms were obtained: type I—900 g, type II—930 g, type I—900 g. 

After the treatment, the products were placed in food containers and were stored for 48 h at 20 °C, and for 48 and 96 h at 4 °C. Time of storage was set according to the time for which fried mushrooms are traditionally stored in these conditions at home, and in restaurant kitchens in Poland. The analysis was conducted on fresh mushrooms as well as on the fried mushrooms before storage and after a storage period of 48 h at 20 °C, and a storage period of 48 and 96 h at 4 °C. For the purposes of the research, parts of mushrooms caps were analyzed along with the sauce formed during frying.

### 2.2. Analysis of Proximate Composition

The samples were analyzed for chemical composition using the AOAC procedures [10]. Moisture was analyzed after drying in 105 °C until reaching the final mass (AOAC No. 930.04). The crude protein (N × 4.38; N, nitrogen) was estimated by the Kjeldahl method (AOAC No. 978.04), the crude fat was determined by extracting a sample with diethyl ether in a Soxhlet apparatus (AOAC No. 920.39) and the ash content was determined by incineration at 460 °C (AOAC No. 920.05). Total carbohydrates were calculated by difference [1]). Energy was calculated according to the following equation: Energy (kcal) = 4 × (g protein) + 3.75 × (g carbohydrate) + 9 × (g fat) [11]. All samples were analyzed in four replications.

### 2.3. Analysis of Antioxidant Properties

Total polyphenols contents and total flavonoids contents was determined in 80% methanol extracts acidified with 0.5% HCl by spectrophotometry using the Folin-Ciocalteu reagent [12] and AlCl_3_ and NaNO_2_ solutions [13,14]. The contents of these compounds were expressed as mg of (+)-catechin per 1 g of fresh weight.

Antioxidant activity was determined in 80% methanol extracts prepared, with heat treatment, against the DPPH radical [15] and against ABTS cation radical [16], as well as by the Ferric Reducing/Antioxidant Power method [17]. The value of the antioxidant activity was determined according to the standard curve prepared for Trolox Equivalent for ABTS and DPPH, and to the standard curve prepared for iron ions (II) for the FRAP method. All samples were analyzed in four replications.

### 2.4. Color Analysis

Color measurement was conducted instrumentally according to the CIE system using a MINOLTA CM-3500d by reflection in a 5 cm diameter cell. Based on that measurement, the following parameters were established: L*—color brightness (L* = 0 black, L* = 100 white), a*—green color (a* < 0) red color (a* > 0), b*—blue color (b* < 0) yellow color (b* > 0), C*—color saturation and h*—hue angle. All samples were analyzed in five replications.

### 2.5. Texture Analysis

Texture measurements were conducted with a TA.XT2 texture analyzer from Stable Micro Systems (Haselemere, Surrey, UK) controlled with the XTRA Dimension v 3.7 program. Cutting test was performed using a modified Kramer shear cell with three sharpened blades. Fifty grams of mushrooms were placed in a Kramer shear cell and cut at the speed of 1 mm/s. The obtained data represented maximum force of cutting (N) and work of cutting (mJ) as an area under the graph of force changes during the cutting test. The analysis was repeated six times for each kind of material.

### 2.6. Sensory Analysis

Due to health safety of those conducting it, sensory analysis was only conducted on the fried samples before storage. 

Sensory analysis was conducted according to ISO standards using a 5-point scale (5—excellent, 4—very good, 3—good, 2—bad, 1—very bad) by a panel of 6, all of whom fulfilled the basic requirements as to sensory sensitivity by ISO standards. A panel consisted of six panelists, three women and three men, each between 25 and 50 years old, with an average age of 32 years. Sensory evaluation was conducted in a room fulfilling the requirements for sensory evaluation. The analysis took into account the basic quality descriptors: appearance-color (IF (importance factor) = 2); appearance-desirability (IF = 2); odor intensity (IF = 2); odor desirability (IF = 3); consistency (IF = 4); flavor intensity (IF = 3); and flavor desirability (IF = 4). The descriptor of total sensory analysis for the products was established by using the importance factor for particular quality descriptors. The number of points obtained after dividing the total points for a given product (the product of the marks for particular characteristics and their importance factors) by the sum of the importance factors was accepted as the overall score. 

### 2.7. Microbiological Analysis

An amount of 1.00 ± 0.01 g of fried mushrooms was transferred to a bottle containing 99 mL of sterile distilled water, and vigorously shaken (30 s). Next, serial dilutions of the samples from 10^−4^ to 10^−6^ were prepared in sterile distilled water, from which cultures were carried out in the appropriate media. The yeasts and molds number was determined according to PN-90/A-75052.08 [18], the coli index was evaluated in the LPB broth (with lactose and bromocresol purple), and the number of lactic acid bacteria was assessed according to PN-90/A-75052/07 [19]. The TVC (total viable count) and the amount of spore-forming bacteria (*Bacillus*) were determined according to PN-EN ISO 4833:2004/Ap1:2005 [20] by culturing the sample diluted 1:1000000, respectively before and after 15 min of boiling. All samples were analyzed in three independent replications.

### 2.8. Statistical Analysis

The results of the investigation were analyzed statistically using one-way analysis of variance, based on the *F*-Snedecor test and *t*-Student test and the significant differences were established at α = 0.05. The Statistica 8.0 (Stat-Soft) program (StatSoft, Inc.; Tulsa, OK, USA) was used for statistical calculations. Linear correlation coefficiency between the level of total polyphenols and total flavonoids, and antioxidant activity were established. Linear correlation coefficiency between the results of sensory analysis, and color and texture parameters was also established.

## 3. Results and Discussion

### 3.1. Proximate Composition

The nutritional value of the fried *Lactarius deliciosus* mushrooms is presented in Table 1. Fresh mushrooms contained 90.68% moisture. The dry weight of mushrooms was characterized by a high content of carbohydrates and crude protein at the level of 55% and 25%, respectively, while fat and ash only amounted to 11% and 9%, respectively. The composition of the analyzed mushrooms was comparable to the data available in literature [1,6].

**Table 1:** Proximate composition of *Lactarius deliciosus* culinary products (g per 100 g fresh weight)

Component	Type of product			
	Fresh mushrooms	I Fried unblanched mushrooms	II Fried blanched mushrooms	III Fried unblanched mushrooms with onions and spices
Moisture	90.68 ± 0.73 ^a^	81.59 ± 1.24 ^b^	80.18 ± 0.95 ^b^	81.62 ± 1.01 ^b^
Proteins	2.35 ± 0.06 ^a^	2.38 ± 0.17 ^b^	2.40 ± 0.11 ^b^	2.38 ± 0.09 ^b^
Fat	1.04 ± 0.01 ^a^	9.25 ± 0.02 ^b^	9.21 ± 0.13 ^b^	9.28 ± 0.11 ^b^
Total carbohydrates	5.17 ± 0.09 ^a^	5.84 ± 0.11 ^b^	6.98 ± 0.14 ^b^	5.70 ± 0.07 ^b^
Ash	0.78 ± 0.99 ^a^	0.99 ± 0.04 ^b^	1.04 ± 0.01 ^c^	1.21 ± 0.05 ^d^
Energy (kcal)	38 ± 0 ^a^	114 ± 0 ^b^	119 ± 1 ^b^	114 ± 0 ^b^

Mean value ± sd; ^a–d^ Represent statistical differences (for one component).

Fried *Lactarius deliciosus* mushrooms contained significantly more of dry substance than fresh fruiting bodies by 9.06–10.50 g per 100 g of fresh weight and ten times the amount of fat. A significant increase was also observed in the amount of other components like crude protein, total carbohydrates, and ash, between 1% and 55%, which is the result of water loss during thermal treatment. As a result of this increase in the content of energetic components during frying, the caloric value of the fried products was three times higher than that of fresh fruiting bodies of *Lactarius deliciosus*. No significant differences in the composition of particular product types were observed, except for the total carbohydrate content and ash content. Total carbohydrate content and ash content were highest in type II product and type III product, respectively. The reason for type II product to present a higher level of total carbohydrate content than other culinary products can be the leaching of soluble substances during blanching, which increases the fraction of insoluble carbohydrates, while the dry weight content remains comparable in all of the products. As for the highest content of ash observed in product III, it can be a result of adding onions and spices, which are a better source of ash than mushrooms. Manzi *et al.* [11,21] during cooking of *Pleurotus ssp.*, *Boletus edulis*, and *Agaricus bisporus*, in their own sauces, detected an increase in the content of particular components of dry weight like the crude protein, ash, and total carbohydrates, as well as an increase of the caloric value by 15%–25%. 

### 3.2. Antioxidant Properties

Polyphenols, and especially flavonoids, are compounds of particular importance to human health. In our diet they might provide health benefits due to their ability to reduce agents by donating hydrogen and quenching singlet oxygen [22]. Total polyphenol content and total flavonoid content, and antioxidant activity of the analyzed products prepared with *Lactarius deliciosus* are presented in Table 2. In 1 g of fresh mushrooms, those bioactive compounds were determined at 0.49 and 0.34 mg respectively expressed as (+)-catechin, while antioxidant activity against ABTS, DPPH, and with the FRAP method was 36.3 and 20.1 µmol TE and 32.4 µmol Fe^2+^, respectively. According to Palacios *et al.* [4] the fruiting bodies of *Lactarius deliciosus* contain 15 mg total polyphenols in 1 g of dry weight, expressed as gallic acid, with the dominating presence of homogentistic acid, gallic acid, chlorogenic acid, and gentistic acid. Moreover, the same authors determined the flavonoid content of the mushrooms at 30 mg total flavonoids expressed as catechin, and among eight mushroom species, which were researched, *Lactarius deliciosus* had the highest content of flavonoid compounds. Literature notes large variations in the content of phenol compounds in fruiting bodies of edible mushrooms, including *Lactarius deliciosus*: total polyphenol content in 1 g varies between 0.58 and 17.25 mg. These variations are caused, in particular, by the environmental conditions and by the enzymatic activity of the mushrooms [3,23,24,25]. Literature available indicates that antioxidant activity in four mushroom species of the *Lactarius* family was determined as EC_50_ value in the reaction with DPPH free radical varies between 8.52 and 30.3, and the fruiting bodies of *Lactarius deliciosus* prevent 50% of lipid oxidation and are characterized by a high antioxidant activity determined with the FRAP method in comparison to other mushrooms [3,24,25].

**Table 2:** Antioxidant properties of *Lactarius deliciosus* culinary products (per 1 g fresh weight).

Antioxidant properties	Storage	Type of product			
		Fresh mushrooms	I Fried unblanched mushrooms	II Fried blanched mushrooms	III Fried unblanched mushrooms with onions and spices
Total polyphenol content (mg of (+)-catechnin)	0	0.49 ± 0.01 ^a^	0.50 ± 0.03 ^a^	0.39 ± 0.03 ^b^	0.50 ± 0.00 ^a^
	48 h/20 °C		0.44 ± 0.02 ^c^	0.36 ± 0.01 ^e^	0.53 ± 0.03 ^f^
	48 h/4 °C		0.47 ± 0.01 ^d^	0.36 ± 0.01 ^e^	0.50 ± 0.03 ^a^
	96 h/4 °C		0.46 ± 0.03 ^c^	0.36 ± 0.01 ^e^	0.52 ± 0.02 ^a,f^
Total flavonoid content (mg of (+)-catechin)	0	0.34 ± 0.02 ^a^	0.36 ± 0.02 ^b^	0.29 ± 0.02 ^c^	0.37 ± 0.03 ^b,d^
	48 h/20 °C		0.29 ± 0.02 ^c^	0.34 ± 0.01 ^a^	0.31 ± 0.01 ^e^
	48 h/4 °C		0.38 ± 0.02 ^d^	0.31 ± 0.02 ^e^	0.34 ± 0.02 ^a^
	96 h/4 °C		0.34 ± 0.02 ^a^	0.36 ± 0.02 ^b^	0.33 ± 0.01 ^a^
Antioxidant activity against ABTS (μmol TE)	0	36.3 ± 0.9 ^a^	28.9 ± 0.3 ^b^	15.1 ± 0.4 ^e^	30.4 ± 0.4 ^d^
	48 h/20 °C		26.9 ± 0.7 ^c^	9.2 ± 0.5 ^f^	32.3 ± 0.7 ^i^
	48 h/4 °C		28.3 ± 0.9 ^b^	11.5 ± 0.4 ^g^	27.5 ± 0.5 ^c^
	96 h/4 °C		30.1 ± 0.1 ^d^	14.1 ± 0.3 ^h^	32.8 ± 0.9 ^i^
Antioxidant activity against DPPH (μmol TE)	0	20.1 ± 0.9 ^a^	12.6 ± 0.9 ^b^	12.1 ± 0.5 ^b^	14.5 ± 0.6 ^c^
	48 h/20 °C		14.0 ± 0.7 ^c^	11.2 ± 0.9 ^d^	15.4 ± 0.8 ^f^
	48 h/4 °C		14.0 ± 0.8 ^c^	11.5 ± 0.6 ^d^	13.7 ± 0.6 ^c^
	96 h/4 °C		13.2 ± 0.9 ^b^	9.8 ± 0.6 ^e^	14.2 ± 0.9 ^c^
Ferric reducing/antioxidant potential—FRAP (μmol Fe^2+^)	0	32.4 ± 1.7 ^a^	22.0 ± 0.5 ^b^	22.1 ± 0.7 ^b^	23.5 ± 1.0 ^b^
	48 h/20 °C		22.2 ± 0.5 ^b^	23.8 ± 2.1 ^b,c^	25.1 ± 1.3 ^c^
	48 h/4 °C		23.8 ± 1.2 ^b,c^	22.0 ± 1.9 ^b^	23.4 ± 0.8 ^b,c^
	96 h/4 °C		25.1 ± 1.4 ^c^	21.2 ± 1.1 ^b,d^	24.0 ± 1.0 ^b,c^

Mean value ± sd; ^a–i^ Represent statistical differences (for one parameter).

During frying of the *Lactarius deliciosus* a decrease in total polyphenol content, total flavonoid content, and antioxidant activity was observed in type II product by 20%, 15%, and by 42%–68%, depending on the method, respectively. In both type I and type III products, the total polyphenol content did not change while the total flavonoid content increased by 6%–9%. At the same time antioxidant activity of those products decreased by 15%–20% against ABTS radicals, by 28%–37% against DPPH radicals and by 27%–32% in the FRAP method. Polyphenol compounds are sensitive to high temperature which may lead to a decrease in polyphenol content during thermal treatment [8]. Thermal treatment in vegetables leads to a loss in total polyphenols of 0% up to 70%; flavonoids are generally more stable than other polyphenol compounds [8,9,26,27]. In addition, a decrease in antioxidant activity may occur during thermal treatment of vegetables, consisting of cooking or frying in a small amount of oil, and it varies from 0% to 95% depending on the material as well as the method and the duration of thermal treatment [8,9]. Thermal treatment not only results in a decrease of the total polyphenol content but also changes their structure, which affects their antioxidant activity. Some of the authors [8,26,27,28] in their research of thermal treatment, particularly stir-frying, have not reported a decrease, or have even reported an increase in polyphenol content. This can be attributed to changes occurring in the polyphenol compounds’ structure, particularly to hydrolysis, which can lead to an increase in reactivity with Folin-Ciocalteu reagent. It is also the result of rapid inactivation of phenol oxidase caused by thermal treatment and, consequently, of inhibition of enzymatic oxidative changes. During thermal treatment a decrease can be observed in the content of other antioxidant compounds, including vitamins [8]. At the same time, new antioxidant substances are produced, namely the products of Maillard reactions [8,9]. As a result of using canola oil rich in tocopherols, fried culinary products can be characterized by a higher antioxidant activity than cooked culinary products [29]. In type II, culinary product losses of antioxidant compounds and decrease of antioxidant activity was not only the effect of thermal degradation, but was also caused by leaching during blanching. At the same time, the highest levels of antioxidants and antioxidant activity was determined in type III product, which can be explained by the fact that onions and spices, rich sources of polyphenols and other antioxidant compound, were added.

During storage of fried *Lactarius deliciosus* their total polyphenol content, total flavonoid content, and antioxidant activity underwent further fluctuations. After the storage period, the total polyphenol content increased by between 0% and 24%, and the total flavonoid content decreased by between 0% and 19%, in comparison to freshly fried products. In the antioxidant activity the most important changes, between −39% and +6% were observed with the ABTS method, while the DPPH and FRAP methods revealed changes between −14% and +19%. More important changes in antioxidants and antioxidant activity were observed in products stored at room temperature for 48 h than in products stored in cooling conditions for 96 h, particularly type II product. Storing processed vegetables (canned or frozen) also results in significant changes in antioxidant activity, or even its complete disappearance, after an eight-month storage period [30].

A high positive correlation coefficiency was observed between the content of total polyphenols and level of antioxidant activity against ABTS radicals (*r* = 0.94) and against DPPH (*r* = 0.67). The correlation coefficient (*r*) between antioxidant activity and total flavonoids, and between FRAP antioxidant activity and total polyphenols, was below 0.5 which shows lack of relationship between those parameters. 

### 3.3. Color Analysis

Table 3 presents the values of the color parameters in the analyzed products prepared with *Lactarius deliciosus*. Immediately after frying, they were characterized by brightness expressed as the L* parameter at the level of 43.40–46.83, which was higher than fresh mushrooms by 2.75–6.18. In fresh mushrooms, the presence of a red color was expressed as the positive value of parameter a*, frying however led to shifting of the red shades surface towards the green shades surface, expressed as the decrease in the value of parameter a* by 14%–18%. The fresh mushrooms as well as the products prepared with them indicated a presence of the color yellow; no significant shift of color surface or decrease of parameter b* were observed during frying. At the same time, frying resulted in a decrease in the value of parameter C* by 4%–12% and in a decrease in the value of parameter h* by 3%–5%. The most important changes in the value of parameter L* were observed in type II product, in turn type III product revealed the most important changes in the value of the other color parameters. During storage further changes in the values of the color parameters occurred. After the storage period the values of parameters L*, a*, b* and C* in comparison to fried mushrooms before storage decreased by, respectively, 1%–8%, 3%–30%, 0%–16% and 0%–18%, while the value of parameter h* increased by 0%–5%. It was noted that type II product revealed the most important changes of the color parameters after storage in comparison to other types of products; it was also noted that more important changes occurred during a 96 h storage period in cooling conditions than during a 48 h storage period at room temperature.

**Table 3:** Color of *Lactarius deliciosus* culinary products.

Parameter	Storage	Type of product			
		Fresh mushrooms	I Fried unblanched mushrooms	II Fried blanched mushrooms	III Fried unblanched mushrooms with onions and spices
L*	0	40.65 ± 0.47 ^a^	45.84 ± 0.68 ^b^	46.83 ± 0.79 ^d^	43.40 ± 0.23 ^c^
	48 h/20 °C		43.40 ± 0.37 ^c^	46.25 ± 0.44 ^b,d^	42.93 ± 0.39 ^c^
	96 h/4 °C		42.86 ± 1.24 ^c^	46.21 ± 0.79 ^b,d^	43.08 ± 0.25 ^c^
a*	0	13.62 ± 0.44 ^a^	11.72 ± 0.52 ^b^	13.06 ± 0.64 ^d^	11.25 ± 1.14 ^b^
	48 h/20 °C		9.90 ± 0.23 ^c^	10.82 ± 0.32 ^e^	10.96 ± 0.18 ^e^
	96 h/4 °C		10.18 ± 0.39 ^c^	9.16 ± 0.11 ^f^	10.04 ± 0.29 ^c^
b*	0	28.68 ± 0.85 ^a^	28.35 ± 1.26 ^a^	29.73 ± 0.20 ^a,c^	27.29 ± 2.21 ^a,d^
	48 h/20 °C		25.88 ± 0.48 ^b^	27.86 ± 0.34 ^a,d^	27.64 ± 0.53 ^a,d^
	96 h/4 °C		24.28 ± 1.28 ^b^	24.85 ± 0.32 ^b^	25.41 ± 0.60 ^b^
C*	0	33.76 ± 0.85 ^a^	30.68 ± 1.36 ^b^	32.48 ± 0.41 ^a^	29.52 ± 2.46 ^d^
	48 h/20 °C		27.71 ± 0.53 ^c^	29.89 ± 0.42 ^d^	29.73 ± 0.53 ^d^
	96 h/4 °C		26.33 ± 1.32 ^c^	26.48 ± 0.30 ^c^	27.32 ± 0.65 ^c^
h*	0	64.59 ± 0.82 ^a^	67.53 ± 0.11 ^b^	66.29 ± 0.95 ^d^	67.61 ± 0.72 ^b^
	48 h/20 °C		69.06 ± 0.14 ^c^	68.77 ± 0.40 ^c^	68.38 ± 0.37 ^c^
	96 h/4 °C		67.25 ± 0.53 ^b^	69.75 ± 0.31 ^c,e^	68.45 ± 0.25 ^c^

Mean value ± sd; ^a–f^ Represent statistical differences (for one parameter).

Color changes in mushrooms occur not only during culinary treatment, but also during other processes like the production of canned foods. According to Zivanovic *et al.* [31], during thermal treatment of the fruiting bodies of *Agaricus bisporus*, the mushrooms become darker, which is a result of non-enzymatic oxidative changes in the polyphenol compounds. At the same time, thermal treatment increases the hydrolysis of proteins and carbohydrates, thus causing the formation of substances, which can participate in carbonyl-amine reactions and contribute to discoloration of the product. Changes in color during frying are also the result of a Maillard reaction, which causes the product to become darker and the color surface to shift towards shades of brown [32,33]. 

A high positive correlation coefficiency was observed between the value of L* parameter and total polyphenols (*r* = 0.68) and level of antioxidant activity measured by three methods (*r* values between 0.78 and 0.88). In addition, the values of a*, C*, and h* parameters showed positive correlation with antioxidant activity measured with the DPPH and FRAP methods (*r* = 0.50–0.73). The correlation coefficient between values of parameters a*, b*, C*, and total polyphenols and antioxidant activity against ABTS, and also values of b* parameter and total polyphenols and antioxidant activity was below 0.50, which shows lack of relationship between those parameters.

### 3.4. Texture Analysis

**Figure 1:**
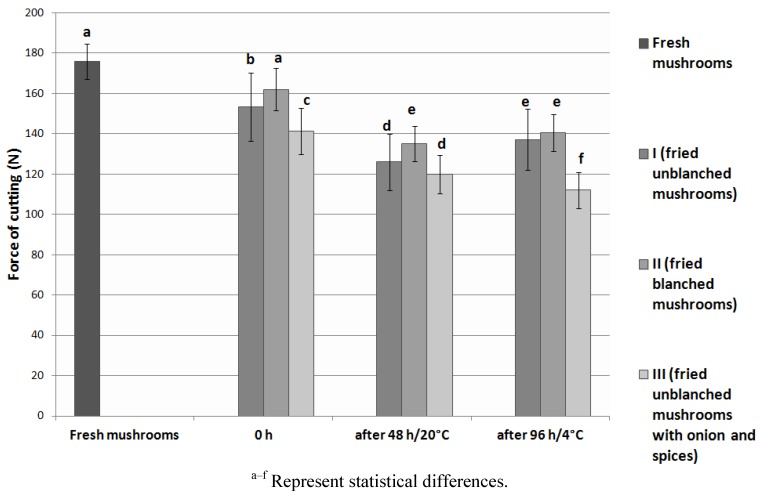
Texture of *Lactarius deliciosus* culinary products–force of cutting (N).

**Figure 2:**
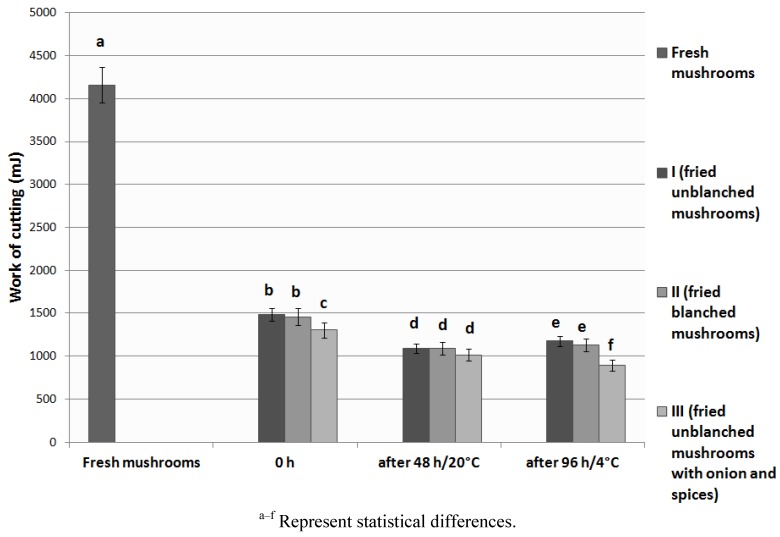
Texture of *Lactarius deliciosus* culinary products–work of cutting (mJ).

The values of texture parameters of the analyzed fried *Lactarius deliciosus* are presented in Figure 1, Figure 2. The force of cutting of fresh mushrooms was 176 N, and decreased by 8%–20% after frying. The work of cutting of fresh mushrooms was 4158 mJ, and it was three times smaller in culinary products. During storage, a further decrease was observed in texture parameters, by between 13% and 21% in the force of cutting, and by between 21% and 31 % in the work of cutting. The smallest values of texture parameters were determined in type III product, while the highest values were determined in type II product, in terms of force of cutting, and in type I product, in terms of work of cutting. Storing the fried mushrooms in cooling conditions resulted in decreasing the values of texture parameters in comparison to type I and type II products stored in room temperature, and the opposite occurred in type III product. In the analyzed fresh and fried *Lactarius deliciosus* mushrooms the texture parameters were characterized by a high standard deviation (5%–15%). This phenomenon often occurs in mushrooms and is caused by the heterogeneous structure of individual mushrooms that form the whole product [34]. Thermal treatment influences the tissue of the mushrooms. The membranes denaturize and their permeability increases, which in turn leads to a loss of water and softening of the cells and causes a change in their structure. The effect of the thermal treatment is more visible in mushrooms than in vegetables because the mushrooms’ cell wall, composed of glucan and chitin, does not offer the support the plant cell wall offers. Moreover, the macrostructures of the mushroom cells undergo hydrolysis, causing further tissue relaxation [31,34,35]. Higher values of force of cutting of unblanched fruiting bodies can be explained by the fact that, during blanching of the mushroom their force for puncturing increases. This is the result of an increase in the fraction of alcohol-insoluble solids in the composition of mushroom cells in relation with the decrease in water content and soluble substances. The shrinking of the cells and the reduction of intercellular spaces causes tighter organization of hyphae in the tissue [36].

### 3.5. Sensory Analysis

**Figure 3:**
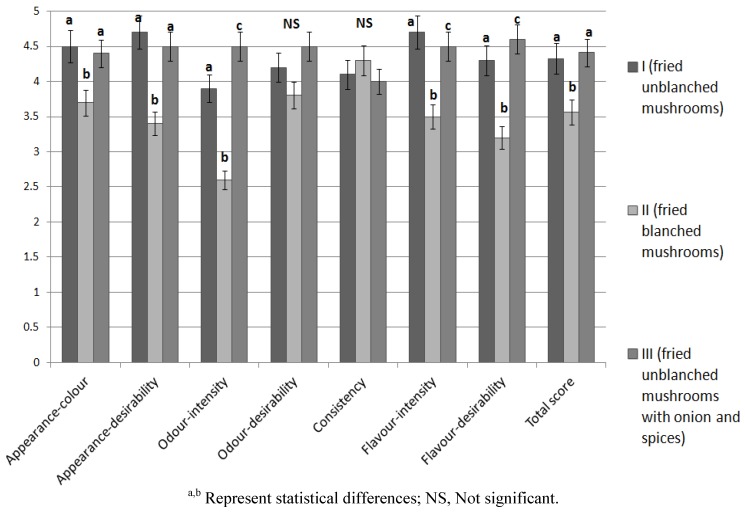
Sensory analysis of *Lactarius deliciosus* culinary products.

The results of the sensory analysis of products prepared with *Lactarius deliciosus* are presented in Figure 3. The highest total score of 4.4 was accorded to type III product prepared with unblanched mushrooms with spices, and type I product scored less by a 0.1 point, which does not constitute a significant difference. In type II product, prepared with blanched mushrooms, the total score was below good. It can be a result of the leaching of aromatic and flavoring substances during blanching [37]. Among the assessed parameters contributing to the total score, the most influential were color, aroma intensity, flavor intensity, and flavor desirability. A high correlation was observed between the sensory analysis of color and consistency, and the results of instrumental analysis of color and texture. The coefficient was particularly high in the case of sensory analysis of color and the color parameter a* (*r* = 0.98) and parameter b* (*r* = 0.71), as well as between consistency and force of cutting (*r* = 0.92).

### 3.6. Microbiological Analysis

Microbiological safety for consumption of edible mushroom has recently been a very popular matter. Venturini *et al.* [38] determined in their research that mushrooms can carry many microorganisms. Fried *Lactarius deliciosus* after a 48 h storage period at room temperature were characterized by a TVC of over 10^6^ (Table 4). At the same time, the samples tested positive for lactic acid bacteria at the level of 10^4^–10^6^. This amount of microorganisms is not permitted in any food product according to the Regulation of the Polish Ministry of Health, dated December 27, 2000. In type I and type III products stored in cooling conditions for a period of 96 h, microorganisms were not detected, and in type II product TVC and the level of lactic acid bacteria did not exceed 10^4^. In terms of microbiological safety it is recommended to keep fried mushrooms in cooling conditions. The addition of spices, which contain antibacterial substances [39], can positively influence the contamination of the final product. It can be assumed that the presence of spices in type III product stunted the development of lactic acid bacteria.

**Table 4:** Microbiology of *Lactarius deliciosus* culinary products after storage.

Type of product	TVC	Yeast and molds	Lactic acid bacteria	*E.coli*	*Bacillus*
After 48 h in 20 °C					
I	+++	Ø	++	Ø	Ø
II	+++	Ø	++	Ø	Ø
III	+++	Ø	Ø	Ø	Ø
After 96 h in 4 °C					
I	Ø	Ø	Ø	Ø	Ø
II	+	Ø	+	Ø	Ø
III	Ø	Ø	Ø	Ø	Ø

Ø, No growth; +, Below 10^4^ cfu per g; ++, Between 10^4^–10^6^ cfu per g; +++, Over 10^6^ cfu per g.

## 4. Conclusions

Fried *Lactarius deliciosus* mushrooms were characterized by a level of moisture lower than fresh mushrooms, and higher of other constituents such as fat, crude protein, total carbohydrates, and ash. Frying in oil led to a decrease of antioxidant activity against ABTS, DPPH, and in FRAP method, however, the level of total polyphenols and total flavonoids remained on a level comparable to fresh mushrooms. In addition, during frying, quality parameters such as color and texture changed. Based on the level of quality parameters and sensory assessment, the conclusion can be drawn that optimal culinary treatment consist of frying unblanched *Lactarius deliciosus* caps, preferably with onion and spices. Due to microbiological safety it is advised to store culinary products in cooling conditions.
